# Maitake mushroom extract in myelodysplastic syndromes (MDS): a phase II study

**DOI:** 10.1007/s00262-014-1628-6

**Published:** 2014-10-29

**Authors:** Kathleen M. Wesa, Susanna Cunningham-Rundles, Virginia M. Klimek, Emily Vertosick, Marci I. Coleton, K. Simon Yeung, Hong Lin, Stephen Nimer, Barrie R. Cassileth

**Affiliations:** 1 Integrative Medicine Service, Memorial Sloan Kettering Cancer Center, 1429 First Avenue, New York, NY 10021 USA; 2 Hematology/Oncology, Weill Medical College of Cornell University, New York, NY USA; 3 Sylvester Comprehensive Cancer Center, Miller School of Medicine, University of Miami, Miami, FL USA; 4 Leukemia Service, Memorial Sloan Kettering Cancer Center, New York, NY USA; 5 Epidemiology & Biostatistics, Memorial Sloan Kettering Cancer Center, New York, NY USA

**Keywords:** Myelodysplastic syndrome, Maitake, Beta-glucan, Infections, Neutrophil, Monocyte

## Abstract

**Background:**

Myelodysplastic syndromes (MDS) are characterized by ineffective erythropoiesis with dysplastic bone marrow leading to peripheral cytopenia, risk of infection, and progression to acute myelogenous leukemia. Maitake mushroom beta-glucan, a dietary supplement, stimulates hematopoietic progenitor cell differentiation, granulocyte colony-stimulating factor production, and recovery of peripheral blood leukocytes after bone marrow injury. This phase II trial examined the effects of Maitake on innate immune function in MDS.

**Methods:**

Myelodysplastic syndromes patients with International Prognostic Scoring System Low- and Intermediate-1-risk disease received oral Maitake extract at 3 mg/kg twice daily for 12 weeks. Primary endpoints included neutrophil count and function tested as endogenous or stimulated neutrophil production of reactive oxygen species (ROS) by flow cytometry compared with age-matched healthy controls (HC). ROS activators were *Escherichia* *coli*, phorbol ester, and the bacterial peptide N-formylmethionyl-leucyl-phenylalanine (fMLP). Complete blood counts, chemistry panels, iron studies, and monocyte function were evaluated.

**Results:**

Of 21 patients enrolled, 18 completed the study and were evaluable. Maitake increased endogenous (basal) neutrophil (*p* = 0.005) and monocyte function (*p* = 0.021). Pre-treatment monocyte response to *E. coli* was reduced in MDS patients compared with HC (*p* = 0.002) and increased (*p* = 0.0004) after treatment. fMLP-stimulated ROS production response also increased (*p* = 0.03). Asymptomatic eosinophilia occurred in 4 patients (*p* = 0.014). Other changes in albumin, hemoglobin, and total protein were not clinically relevant.

**Conclusions:**

Maitake was well tolerated. Enhanced in vitro neutrophil and monocyte function following treatment demonstrate that Maitake has beneficial immunomodulatory potential in MDS. Further study is warranted.

## Introduction

Myelodysplastic syndromes (MDS) are a heterogeneous group of clonal bone marrow stem cell disorders characterized by ineffective erythropoiesis with dysplastic bone marrow leading to peripheral blood cytopenias and a variable propensity for progression to acute myelogenous leukemia (AML). Neutropenia occurs in 50 % of newly diagnosed patients, more frequently in those with higher-risk (70–80 %) compared with lower-risk (15–20 %) MDS [1]. Pneumonia was identified as the leading cause of death in a recent investigation of untreated MDS patients with low or intermediate risk. The pathogen was mainly bacterial although sometimes fungal or more rarely viral [2]. The use of hypomethylating agents and lenalidomide in MDS patients will transiently worsen neutropenia and has stimulated investigation into the causes of infection and strategies to reduce risk [1].

Infection has been largely attributed to neutrophil dysfunction rather than neutropenia, since early studies showed that neither infections nor mortality were related to low neutrophil count [3]. A recent study in Asian MDS patients using the French–American–British (FAB) classification and International Prognostic Scoring System (IPSS) confirmed that neutrophil count did not predict prognosis in persistent severe neutropenia (neutrophil count < 500 μ/L) and that even a neutrophil count <200 μ/L showed only borderline prognostic significance [4]. Early studies reported that granulocyte function (mainly neutrophils in circulation) was reduced across FAB classifications [5]. Subsequent investigations revealed variable impairment in different neutrophil functions independent of neutrophil number, including reduced chemotaxis, decreased adhesion molecule expression, and loss of microbicidal activity, especially in patients with recurrent infection [6, 7]. Fuhler et al. identified mechanisms of neutrophil dysfunction in MDS as disturbed chemokine receptor-induced response and impaired migration of circulating neutrophils toward the chemoattractant interleukin-8 (IL-8). Impaired migration in blood could be traced to defects in early hematopoietic progenitors in bone marrow [8, 9].

Intracellular generation of highly reactive oxygen species (ROS) in the respiratory burst pathway is produced by the reduced nicotinamide-adenine dinucleotide phosphate (NADPH) oxidase complex. ROS activation is triggered by receptor-mediated binding of soluble chemotactic agents, such as the bacterial peptide N-formyl-methionyl-leucyl-phenylalanine (fMLP) and receptor-independent phorbol ester phorbol-myristate-acetate (PMA). Granulocyte colony-stimulating factor (G-CSF), granulocyte–macrophage CSF (GM-CSF), and cytokines prime the ROS response. Early studies showed that ROS activity varied in MDS patients from normal to abnormal independent of neutrophil number [10–12]. Priming with G-CSF and GM-CSF could partially enhance fMLP-stimulated ROS activity in MDS patients’ neutrophils compared with healthy control responses [13]. Subcutaneous administration of GM-CSF to MDS patients enhanced neutrophil function, assessed ex vivo as microbicidal activity [14]. G-CSF treatment of high-risk MDS patients showed a borderline protective effect against infections [15]. However, use of GM-CSF and G-CSF treatment in MDS has been limited due to concerns about effects on progression, safety, and side effects [16].

We recently reported that oral administration of Maitake beta-glucan, a dietary supplement, stimulated hematopoiesis in bone marrow, recovery of circulating monocyte/neutrophil numbers, and normalized ROS production compared with no treatment or G-CSF-treatment in a mouse model of acute hemotoxic bone marrow injury [17]. Since Maitake beta-glucan induced G-CSF in human umbilical cord blood monocytes stimulating colony-forming unit-granulocyte/macrophage (CFU-GM) differentiation in circulating hematopoietic progenitors [18], we hypothesized that Maitake would be effective in MDS.

Maitake extract, derived from the fruit body of the edible mushroom *Grifola frondosa,* contains beta-glucans with a 1,6-glucan main chain and 1,3-branches [19]. Maitake beta-glucan does not show direct cytotoxic or cytocidal activity, but inhibited lung metastasis when given by intraperitoneal injection (i.p.), enhancing IL-12 production and activating natural killer cells [20]. Maitake increased messenger ribonucleic acid expression of GM-CSF, G-CSF, M-CSF, interferon, and IL-12 p40, and attenuated decrease in CFU-GM colonies of cisplatin-treated mice [21]. Maitake had dose-dependent hematopoietic effects on mouse bone marrow cells in vitro, protecting CFU-GM progenitor cells from doxorubicin toxicity [22]. Ito et al. [23] recently showed that Maitake enhanced granulopoiesis and mobilized granulocytes and progenitors by stimulating G-CSF production in cyclophosphamide-induced granulocytic mice. Lin et al. [24] showed that oral Maitake stimulated homing and engraftment of transplanted donor cord blood cells into recipient mice, while Ito et al. [23] demonstrated that Maitake administered i.p. caused downregulation of chemokine receptor CXCR4, and the ligand stromal-derived factor-1 in the bone marrow of granulocytic mice, causing granulocyte mobilization. Maitake appears to enhance differentiation and migration of hematopoietic cells including progenitors and thereby enhances peripheral myeloid cell ROS function.

In our previous dose-escalation trial, breast cancer patients receiving Maitake extract orally at 5–7 mg/kg daily over 3 weeks showed significant dose-related changes in immune function with no serious adverse events or dose-limiting toxicity [25]. Based on these dose effects, the present study was launched using Maitake extract at 6 mg/kg (i.e., 3 mg/kg twice daily), to assess neutrophil and monocyte function in MDS patients.

## Methods

### Patients

This phase II, open-label, non-randomized, safety, and efficacy trial enrolled MDS patients with IPSS low- or intermediate-1-risk disease who met criteria for MDS based on the FAB and World Health Organization classification systems [26, 27]. Additional eligibility criteria included age ≥18 years, ability to sign informed consent, bone marrow blasts ≤10 %, absolute neutrophil count (ANC) ≥0.5 K/mcL, and stable disease without history of recurrent infections, treatment with a hypomethylating or other disease-modifying agent, or prior stem cell transplant. Exclusion criteria included history of AML, known human immunodeficiency virus (HIV) infection or allergy to mushrooms. The Memorial Sloan Kettering Cancer Center (MSKCC) Institutional Review Board approved the study. Patients were enrolled after informed consent was obtained by the Leukemia Service at MSKCC.

### Endpoints

Primary efficacy endpoints were ANC and neutrophil function as measured by changes in respiratory burst response. Changes in neutrophil count were described using the International Working Group (IWG) modified response criteria for MDS [28].

Secondary efficacy endpoints included changes in hemoglobin, platelet, and reticulocyte counts; G-CSF and GM-CSF levels, monocyte function as measured by respiratory burst response, and iron studies in part because beta-glucans are susceptible to free-radical degradation [29].

Safety was assessed with serial blood chemistry panels and symptom assessment, performed at baseline and study visits at weeks 1, 3, 7, 9, and 12. Adverse events were summarized by grade defined by Common Terminology Criteria for Adverse Events (CTCAE) version 4.0 [30].

### Study protocol

Following double baseline evaluation (1 week apart) for hematologic parameters, immune function studies, and symptom assessment, patients were instructed to take Maitake extract (3 mg/kg) twice daily by mouth for 12 weeks [25]. Symptom assessment, hematologic parameters, and immune studies were performed at two baseline visits and during treatment weeks 1, 3, 7, 9, and 12. Measures specific only to the first baseline visit included complete blood count and reticulocyte counts, iron status (serum iron, ferritin, and total iron-binding capacity), and chemistry panel. Iron status and chemistry panel studies were repeated at week 7 and week 12. Blood samples from healthy volunteers were assessed in parallel with patients’ immune studies over the 2-year study period. Data collected over the 12-week period of Maitake consumption were compiled and compared with baseline studies.

### Healthy controls (HC)

We compared the results of immune function studies in the MDS patients to age-matched healthy volunteers, to control for the possibility of waning age-related immune function in our MDS cohort (median age, 70). Volunteers were recruited through study flyers with assistance from the MSKCC volunteer office. Inclusion criteria were healthy individuals ≥55 years of age. Exclusion criteria were current use of corticosteroids or other immunosuppressants, known history of HIV infection, current or previous history of malignant disorder except adequately treated non-melanoma skin cancer, curatively treated in situ cancer of the cervix, or other solid tumors curatively treated with no evidence of disease for >3 years.

### Intracellular ROS activity

Neutrophil and monocyte function was measured on freshly drawn anticoagulated blood from patients and volunteers in the Weill-Cornell Cellular Immunology laboratory. Endogenous and activated intracellular ROS production was assessed by flow cytometry as previously described [17, 31]. Activators were as follows: opsonized *Escherichia* *coli*, a physiologic, whole bacterial particulate stimulus that signals through Toll-Like Receptor 4, the lipopolysaccharide (LPS) receptor; PMA, a membrane-soluble signal that bypasses receptor binding, measures capacity for ROS production, and requires an intact NADPH oxidase system; and fMLP. Aliquots of heparinized whole blood from study subjects and HC were mixed with or without activators (Phagoburst, Orpegen Pharma, Heidelberg, Germany) and briefly incubated at 37 °C. Addition of the fluorogenic substrate dihydrorhodamine (DHR-123) was used to detect formation of reactive oxidants after conversion to rhodamine-123. Red cells were removed by lysis and cells were partially fixed. DNA stain was added. Flow cytometric evaluation of respiratory burst activity was performed (FACSCalibur, Becton–Dickinson, San Jose, California), using CellQuest software for acquisition and FlowJo software (Tree Star, Ashland, Oregon) for analysis of the percentage of gated granulocyte and monocyte populations producing ROS and relative amounts by geometric mean fluorescence in each sample.

### Serum GM-CSF and G-CSF

Blood samples were collected at each visit, and serum was obtained and frozen at −80 °C in multiple aliquots for each patient pending analysis by enzyme-linked immunosorbent assay. Due to a freezer malfunction, all the samples were inadvertently thawed and were not evaluated.

### Maitake extract

Maitake mushroom (*G. frondosa*) liquid extract was prepared from raw mushroom cultivated in controlled facilities (Yukiguni Maitake Company Ltd., Japan). After harvesting, the fruiting body was extracted with hot water and alcohol and dried to powder form (Nanba et al. [19], U.S. Patent # 5,854,404) and tested for contaminants including endotoxin (*Limulus amebocyte* lysate assay). Glycosyl composition analysis [32] and stimulation of G-CSF production were used as quality control markers [18]. The powder was dissolved in glycerin for liquid formulation to yield a concentration of 40 mg/mL (Yellow Emperor Inc., Eugene, Oregon) via the distributor (Tradeworks Group, Brattleboro, Vermont). Manufacturing and control methods were filed with the US Food and Drug Administration under Investigational New Drug #68853.

### Statistical methods

The sample size was based on published data of neutrophil count, 1,200/mm^3^ (SD 600/mm^3^) [33–35]. A study with 20 evaluable patients would have a confidence interval (CI) around the change in neutrophil counts at 12 weeks of ±270/mm^3^. Baseline values for the neutrophil and monocyte count and function tests were calculated as the average of values between first and second baseline assessments. Percent change was calculated as the difference between the value at 12 weeks and baseline, divided by the baseline value and multiplied by 100. A one-sample *t* test was used to evaluate whether the change between raw score at baseline and raw score at 12 weeks was equal to zero.

The protocol suggested the use of analysis of variance (ANOVA) to test whether the means of the baseline neutrophil function tests and means of the baseline monocyte function tests were equal between HC and MDS patients receiving Maitake mushroom extract. However, due to the non-normal distribution in these groups, a rank sum test was used as the principal method of analysis. Although we considered age as a potential confounder, it was not included as a variable in the analysis due to the small sample size.

A subgroup analysis was performed on MDS ROS responses at baseline, weeks 1–3, and weeks 7–12 by repeated measures ANOVA (Prism 5.0). If significant overall variance was found, Tukey’s posttest was used to determine significance of pairwise comparisons. MDS patients’ data were grouped and evaluated as: baseline: repeated values from 2 pre-treatment studies; weeks 1–3: repeated values comprising 2 sets of studies at weeks 1 and 3; weeks 7–12: repeated values comprising 3 sets of studies at weeks 7, 9, and 12. For HC, all values were in a single group and data for the 4 patients with 2 studies were treated as repeated values.

## Results

### MDS patient population characteristics

**Fig. 1:**
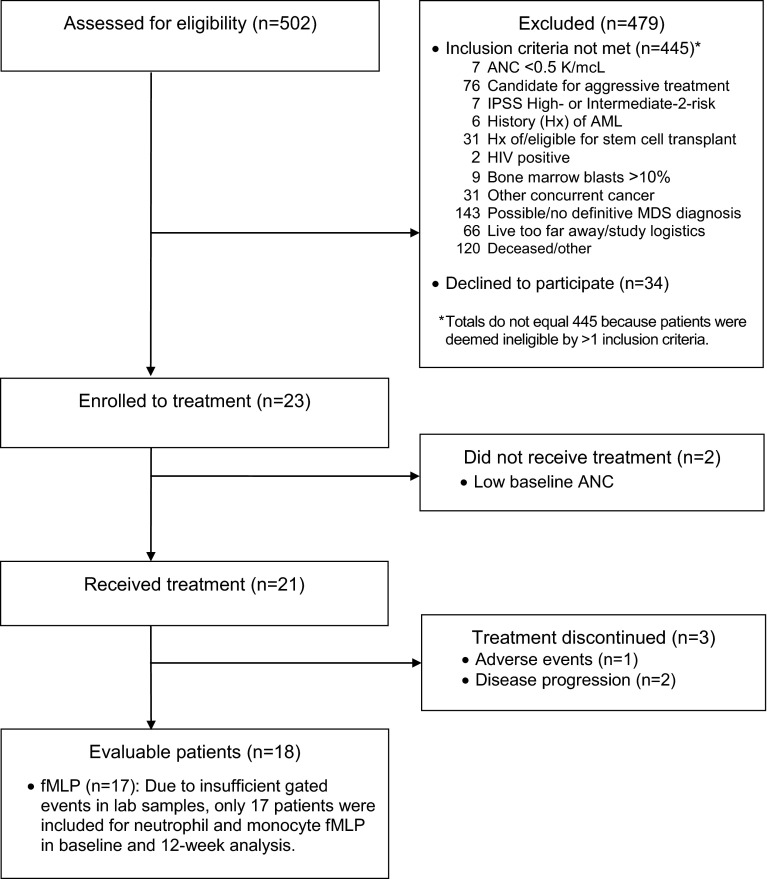
Patient flow diagram, from time of enrollment to completion of study or withdrawal

Between April 2010 and June 2012, 23 patients met eligibility criteria and gave informed consent for the study. Two patients were removed during the double baseline screening period for having ANC values ≤0.5 at the second screening with an ANC >0.5 at initial screening, and 21 patients received Maitake extract (Fig. 1). Baseline patient characteristics are summarized in Table 1. The median age of 70 years is consistent with MDS diagnosis in the USA [36]. The time between diagnosis and study entry (approximately 2 years) allowed for adequate pre-treatment assessment of disease stability. MDS subtypes are summarized in Table 1.

**Table 1:** Baseline characteristics of evaluable MDS patients (*n* = 18)

Patient characteristics	
Male	11 (61 %)
Age	70 (64, 75)
*Race*	
White	16 (89 %)
Black	1 (6 %)
Other	1 (6 %)
Time from diagnosis to study consent (year)	2.3 (1.4, 4.4)
*ECOG performance status*	
Fully active (0)	9 (50 %)
Restricted from physically strenuous activity (1)	9 (50 %)
*Bone marrow biopsy karyotype*	
Normal	9 (50 %)
11q deletion	2 (11 %)
20q deletion	5 (28 %)
Trisomy 8	1 (6 %)
Trisomy 1	1 (6 %)
*MDS classification*	
RCMD	10 (56 %)
RARS	2 (11 %)
RCMD-RS	2 (11 %)
RCUD	2 (11 %)
CMML	1 (6 %)
RAEB-1	1 (6 %)

Results are presented as median (interquartile range [IQR]) or frequency. Some totals exceed 100 % due to rounding

*CMML* chronic myelomonocytic leukemia, *RAEB*-*1* refractory anemia with excess blasts (EB), -1: cytopenias < 5 % blasts, no Auer rods, < 1 × 109/L monocytes in peripheral blood, *RARS* RA with ringed sideroblasts (RS), *RC* refractory cytopenia, *RCMD* RC with multilineage dysplasia (MD), *RCUD* RC with unilineage dysplasia (UD)

### Patient disposition

A total of 18 patients, 7 women and 11 men, completed the planned 12-week treatment and were evaluable. One patient withdrew due to CTCAE 4.0 grade 1 diarrhea, and 2 other patients were removed due to disease progression. One patient with chronic myelomonocytic leukemia at baseline abruptly progressed to AML after 3 weeks of study medication and expired 2 weeks later. Another patient was removed by the treating physician due to persistent, although not worsening, leukopenia and the decision to try aggressive treatment. Leukopenia preceded Maitake treatment and was evaluated as probably unrelated.

### Toxicity

Maitake was generally well tolerated. CTCAE 4.0 grade 1 eosinophilia was noted in 4 patients, and two of these patients also experienced grade 1 diarrhea. One patient experienced grade 1 nausea. One patient with grade 1 diarrhea withdrew consent due to the persistent, albeit low-grade nature of the diarrhea.

### Baseline pre-treatment hematologic parameters

Compared with the normal range neutrophil values from MSKCC Clinical Laboratories, 14 (78 %) patients were normal and 4 patients had a low ANC (<1.5 K/mcL) at baseline. Most patients had normal levels of monocytes, neutrophils, and lymphocytes, but 10 (56 %) had a low white blood cell (WBC) count (<4 K/mcL). Three patients had low neutrophil counts with normal monocyte and lymphocyte counts; one patient had a low neutrophil count, normal lymphocyte count, and high monocyte count (>1.3 K/mcL); one patient had normal neutrophil and monocyte counts and a low lymphocyte count (<0.5 K/mcL); five patients had a low WBC (<4 K/mcL) with normal neutrophil, monocyte, and lymphocyte counts (Table 2).

**Table 2:** Mean change in complete chemistry panel values between baseline and 12 weeks in evaluable MDS patients, *n* = 18

Test (units)	Mean (SD) at Baseline	Mean (SD) at 12 Weeks	Mean change (95 % CI)	*p* value
Albumin (g/dL)	4.5 (0.2)	4.3 (0.3)	−0.2 (−0.2, −0.1)	0.001
Alkaline phosphatase (U/L)	67.2 (26.1)	68.1 (24.3)	0.9 (−2.5, 4.3)	0.6
Alkaline aminotransferase (U/L)	28.6 (12.8)	26.4 (13.7)	−2.2 (−8.0, 3.5)	0.4
AST (U/L)	29.5 (10.5)	28.1 (13.6)	−1.4 (−5.3, 2.6)	0.5
Basophils (K/mcL) (n = 14)	0.7 (0.4)	0.7 (0.3)	0.0 (−0.1, 0.1)	0.8
Bilirubin (mg/dL)	0.7 (0.4)	0.7 (0.3)	0.0 (−0.1, 0.1)	0.8
BUN (mg/dL)	18.7 (5.0)	19.0 (6.4)	0.3 (−1.9, 2.4)	0.8
Calcium (mEq/dL)	9.4 (0.5)	9.2 (0.5)	−0.2 (−0.4, 0.1)	0.2
Chloride (mEq/L)	106.2 (3.1)	105.7 (2.9)	−0.5 (−2.1, 1.1)	0.5
CO_2_ (mEq/L)	28.9 (2.6)	28.2 (2.1)	−0.7 (−2.0, 0.6)	0.3
Eosinophil (K/mcL) (*n* = 14)	2.5 (1.4)	3.9 (2.4)	1.4 (0.4, 2.5)	0.011
Total iron (mcg/dL)	121.6 (47)	129.8 (68.1)	8.2 (−17.9, 34.3)	0.5
Ferritin (ng/mL)	225.6 (296.5)	192.2 (203.5)	−33.4 (−118.2, 51.4)	0.4
Glucose (mg/dL)	111.2 (47.5)	123.4 (51.1)	12.2 (−1.9, 26.3)	0.086
Hemoglobin (g/dL)	11.5 (1.4)	11.0 (1.7)	−0.5 (−0.8, −0.2)	0.003
Hematocrit	34.1 (4.1)	33.0 (4.7)	−1.1 (−2.0, −0.3)	0.012
Iron-binding capacity (mcg/dL) (*n* = 17)	327.8 (72.8)	328.1 (84.1)	0.2 (−16.8, 17.3)	>0.9
Potassium (mEq/L)	4.5 (0.4)	4.3 (0.2)	−0.1 (−0.3, 0.1)	0.2
Lymphocytes (K/mcL)	32.3 (11.5)	33.8 (12.6)	1.5 (−1.1, 4.1)	0.2
Sodium (mEq/L)	141.6 (1.7)	140.6 (2.0)	−1.1 (−2.3, 0.2)	0.082
Red blood cell count	3.6 (0.7)	3.4 (0.7)	−0.1 (−0.2, 0.0)	0.025
Reticulocytes (%)	2.4 (1.5)	2.3 (1.6)	−0.1 (−0.5, 0.4)	0.8
Total protein (g/dL)	7.2 (0.5)	7.0 (0.5)	−0.2 (−0.3, −0.1)	0.001
Platelet counts (K/mcL)	148.0 (106.9)	146.3 (100.3)	−1.7 (−11.9, 8.4)	0.7
White blood cell count (K/mcL)	3.9 (2.1)	3.7 (2.4)	−0.2 (−0.5, 0.2)	0.4

### Baseline Intracellular ROS activity in MDS patients

Baseline analysis was carried out using pre-treatment samples from 17 patients. Due to insufficient gated events, one MDS patient’s blood sample was excluded. There was no significant difference in neutrophil and monocyte endogenous intracellular ROS production when baseline values of MDS patients were compared with HC (Table 3). ROS response to fMLP and PMA were also comparable for MDS and HC. In contrast MDS patients’ monocytes demonstrated a significantly lower ROS response to *E. coli* stimulation compared with HC (mean difference −14.3; 95 % CI −21.1, −7.4; *p* = 0.002; Table 3).

**Table 3:** Mean (SD) baseline neutrophil and monocyte count and function in evaluable MDS patients and HC and mean change between baseline and 12 weeks in evaluable MDS patients, *n* = 18

	Healthy controls (*n* = 18)	MDS patients at baseline (*n* = 18)	MDS patients at 12 weeks (*n* = 18)	Mean difference between HC and MDS patients at baseline (95 % CI)	*p*	Change from baseline to 12 weeks in MDS patients (95 % CI)	*p*
Neutrophil count	–	2.0 (1.0)	1.7 (1.0)	–	–	−0.3 (−0.5, 0.0)	0.044
*Neutrophil function*							
Unstimulated	5.1 (3)	4.1 (3.1)	7.3 (6.3)	−1.0 (−3.0, 1.0)	0.2	3.2 (1.3, 5.1)	0.005
*E. coli*	95.5 (2.4)	90 (10.1)	93.5 (10.5)	−5.5 (−10.3, −0.8)	0.4	3.5 (−3.7, 10.7)	0.4
fMLP (*n* = 17)	11.8 (5.3)	9.6 (5.0)	12.9 (8.4)	−2.1 (−5.5, 1.3)	0.2	3.2 (−0.5, 7)	0.11
PMA	96.6 (4.3)	94.2 (7.0)	96.8 (5.1)	−2.4 (−6.2, 1.4)	0.2	2.6 (−1.0, 6.2)	0.2
Monocyte count	–	11.4 (9.9)	12.1 (11.3)	–	–	0.7 (−1.4, 2.8)	0.5
*Monocyte function*							
Unstimulated	5.3 (5.4)	2.8 (2.4)	6.7 (6.2)	−2.4 (−5.2, 0.4)	0.2	3.9 (0.9, 6.9)	0.021
*E. coli*	89 (4.2)	74.7 (14.5)	85.1 (14.0)	−14.3 (−21.1, −7.4)	0.002	10.4 (5.9, 14.9)	0.0004
fMLP (*n* = 17)	10.7 (7.3)	9.7 (6.5)	17.9 (13.0)	−1.0 (−5.5, 3.6)	0.7	8.2 (1.5, 14.9)	0.030
PMA	95.9 (4.1)	86.3 (14.9)	91.9 (10.0)	−9.6 (−16.8, −2.5)	0.10	5.7 (0.0, 11.3)	0.068

### Effect of Maitake treatment on hematologic parameters

Most study patients had normal neutrophil counts at baseline. When total neutrophil and monocyte counts were examined over time, no patient met IWG criteria for Hematologic Improvement, although three patients showed transient increases in ANC values that were not sustained. Mean monocyte count (Table 3) was not significantly increased at 12 weeks. A treatment-related decrease in mean neutrophil count (−0.3; 95 % CI −0.5, 0.0; *p* = 0.044) was not clinically meaningful (Table 3). A small significant decrease in mean hemoglobin, correlating with changes in hematocrit and red blood cell (RBC) count, was observed from 11.5 (±1.4) to 11.0 (±1.4) g/dL as shown in Table 2. Mean corpuscular hemoglobin did not decrease (data not shown). No patient became anemic (Hgb < 9 g/dL). The significant increase in mean eosinophil count (1.37 K/mcL; *p* = 0.011) was considered CTCAE Grade 1, while basophil count was unchanged (Table 2).

### Effect of Maitake treatment on ROS activity in MDS patients

**Fig. 2:**
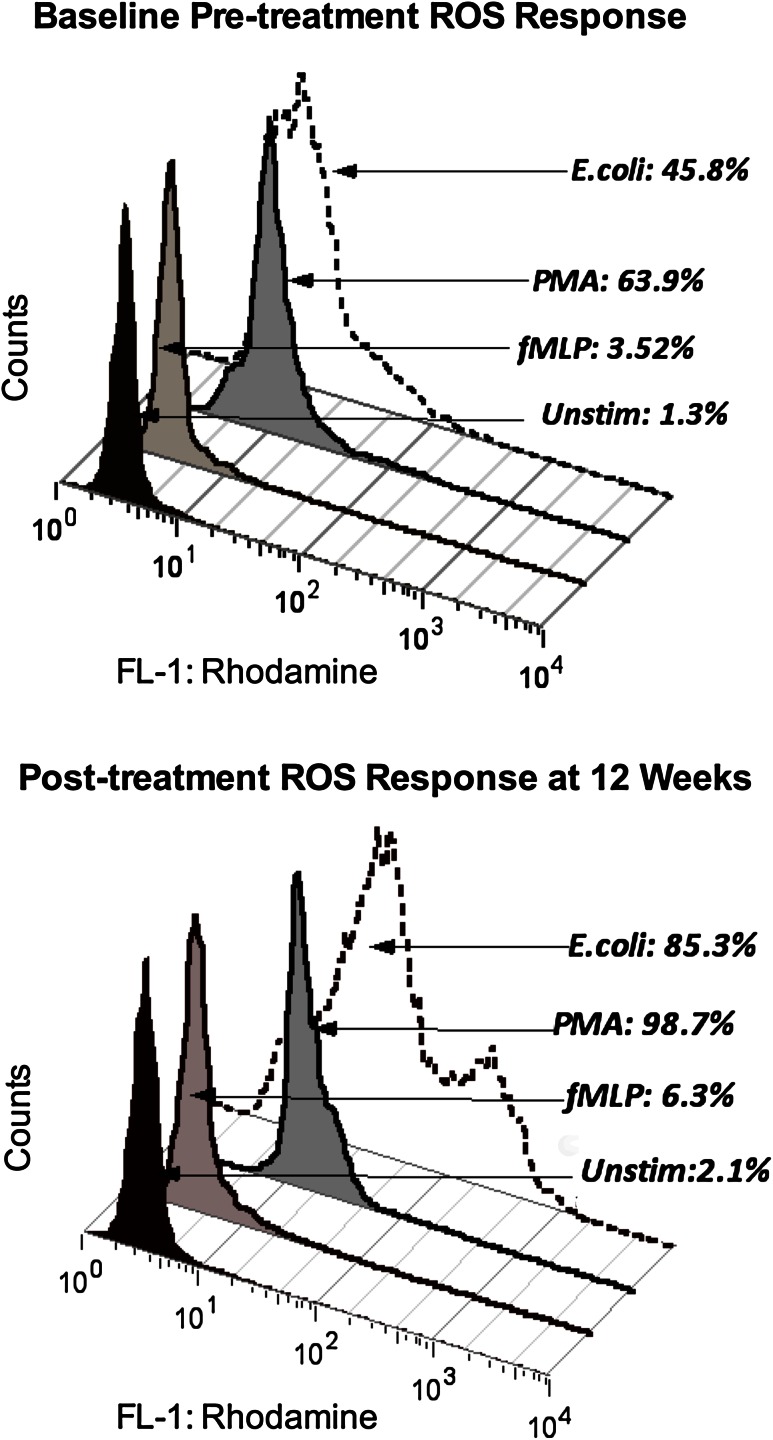
Histogram of pre- and post-treatment monocyte responses. Flow cytometry data for one representative patient shows the percentage of monocytes producing ROS after incubation of peripheral blood with fMLP, opsonized *E. coli*, or PMA compared with the parallel, unstimulated culture assessed ex vivo at baseline and at 12 weeks. Initial gating was performed on populations of monocytes (as distinct from lymphocytes and granulocytes) in the unstimulated cultures using CellQuest software to establish parameters for forward versus side light scatter gating. ROS activity in the unstimulated culture condition was then determined by detection of rhodamine fluorescence in the same physically gated population using the FL-1 channel (fluorescein isothiocyanate, FITC) parameter. Monocyte ROS responses to fMLP, *E. coli*, and PMA on each date were compared with their unstimulated response by detection of FL-1 fluorescence in the comparable physical population defined in the parallel unstimulated culture before and after Maitake treatment using FlowJo software to apply the same gate to the culture set

**Fig. 3:**
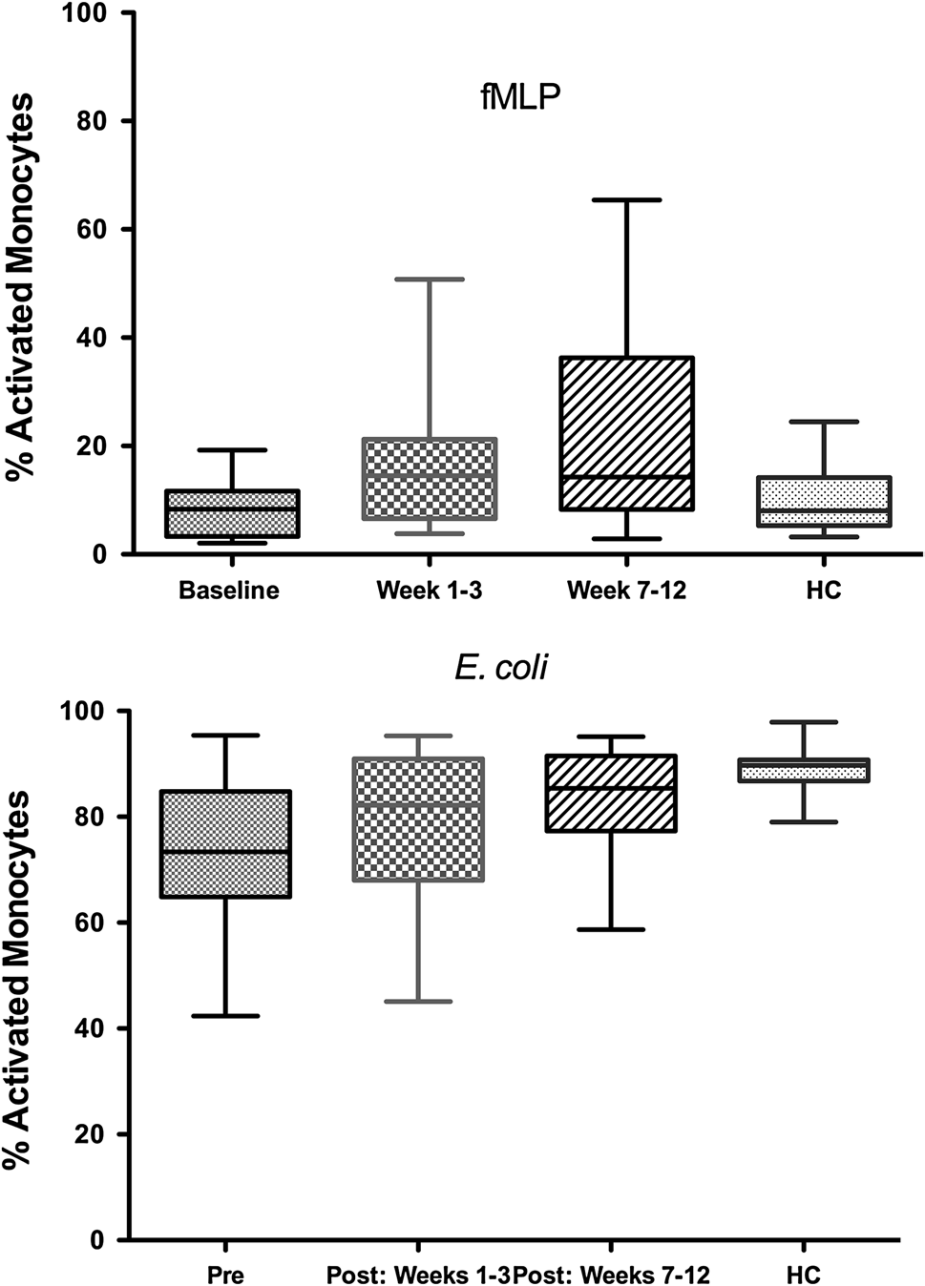
Effect of oral Maitake extract in vivo on respiratory burst activity (ROS) and in vitro in MDS patients. Data show changes in the monocyte ROS function over the study period compared with baseline levels and HC responses. MDS patient monocytes showed increased ROS activity compared with their baseline levels for fMLP (*top panel*) and for *E. coli* (*bottom panel*). Baseline levels were lower in MDS patients compared with HC for *E. coli*, but not different from HC at 7–12 weeks as shown

After 12 weeks of Maitake treatment, MDS patients showed an overall increase in mean endogenous neutrophil ROS production ex vivo, indicating enhanced basal function (3.2; 95 % CI 1.3, 5.1; *p* = 0.005; Table 3). Basal monocyte function also increased at 12 weeks compared with pre-treatment levels (*p* = 0.021). Stimulated ROS production in monocytes increased in response to both *E. coli* (*p* = 0.0004) and fMLP (*p* = 0.03). Response to PMA did not change in either cell type, and neutrophil responses to fMLP and *E. coli* were not affected. These responses were comparable with those of HC at baseline. Figure 2 shows a histogram of pre- and post-treatment monocyte responses to fMLP, PMA, and *E. coli* in a single patient compared with endogenous activity at one baseline visit and again at 12 weeks. Individual MDS patients showed variable changes over the 5 treatment week visits. Figure 3 shows MDS monocyte responses to fMLP and to *E. coli* at pre-treatment baseline visits, at weeks 1–3, and at weeks 7–12 compared with HC (see Methods). MDS patient monocytes stimulated with fMLP showed increased ROS activity at weeks 7–12 compared with baseline (*p* < 0.01) and with HC (*p* < 0.05) by repeated measures ANOVA and Tukey’s posttest. Response to *E. coli* was reduced compared with HC at baseline, but not at 12 weeks (Table 3). Repeated measures by ANOVA and Tukey’s posttest also show an increase at weeks 7–12 in MDS patients compared with baseline (*p* < 0.05).

## Discussion

Infections are the most common cause of death in patients with lower-risk MDS [2]. Although antibacterial and antifungal prophylaxis may be employed, the efficacies are limited [1]. We have previously shown that Maitake beta-glucan promotes maturation of hematopoietic progenitor cells (HPC) in vitro and enhances the recovery of peripheral blood leukocyte numbers and ROS function after chemotoxic bone marrow injury in vivo [17, 18]. The present study examined the effects of oral Maitake extract treatment on peripheral blood neutrophil and monocyte function in untreated, lower-risk MDS patients.

Impaired host defense in MDS is associated with functional defects in the myeloid lineage including HPC and presents as aberrant functioning and has selective effects in host defense mechanisms [9, 12]. Neutrophil and monocyte production of ROS during the respiratory burst is essential for bactericidal activity. Prodan et al. [12] using a similar whole blood flow cytometric assay reported that defects in both monocyte and neutrophil ROS production were characteristic of MDS patients, independent of neutrophil numbers, and worsened with more advanced disease. Fianchi et al. [7] have reported that isolated neutrophils displayed reduced microbicidal activity, explaining the high frequency of recurrent infections in MDS patients who were not neutropenic. They demonstrated progressive loss of microbicidal activity against gram-negative *E. coli*, but not gram-positive lactobacilli or fungus (*Candida albicans)* over the course of monthly studies in patients compared with corresponding functions at diagnosis, suggesting that immune dysfunction in MDS may be pathogen-specific and involve selective host defense pathways.

Our results demonstrate for the first time that monocyte ROS response to physiological *E. coli* is reduced in lower-risk MDS patients, but could be restored after 12 weeks of Maitake treatment. Furthermore, monocyte ROS response to fMLP, the bacterial peptide analogue, was significantly increased. Both neutrophils and monocytes showed increased basal production of ROS after Maitake treatment. Recent studies show that LPS from *E. coli* activates neutrophils to prime monocyte ROS production, leading to release of proinflammatory cytokines and further priming of neutrophils [37, 38]. Our data therefore suggest that monocyte ROS dysfunction may be an early marker of impaired microbicidal activity due to defective monocyte–neutrophil interaction. Maitake has been shown to stimulate human neutrophil phagocytosis in vitro [39].

Endogenous ROS production is necessary to maintain basal activity and is regulated by cytokines and the growth factors GM-CSF and G-CSF. Our observation that MDS patients showed increased neutrophil ROS response after Maitake treatment suggests that G-CSF induction in bone marrow leads to HPC maturation and release of more functionally competent cells [18].

We did plan to measure serum G-CSF (and GM-CSF for comparison) on an exploratory basis but were unable to perform the experiment due to concerns about specimen integrity after freezer malfunction. In our breast cancer trial with oral Maitake beta-glucan [25], we assessed circulating levels of G-CSF in a subset of 6 patients comparing double baseline levels with 3 follow-up visits over a 12-week period. All patients showed positive or elevated G-CSF at baseline visits (11–80 pg/mL). While marked changes occurred in some, differences between baseline and post-treatment were not significant (Cunningham-Rundles and Lin, unpublished data).

Others have reported that ROS are higher in RBCs but not in neutrophils in MDS patients, compared with normal cells. Moreover, increased ROS in RBCs from MDS patients correlated with increased serum ferritin due to iron accumulation [14, 40]. Although serum ferritin levels did not change during our study, it is possible that Maitake does not increase RBC ROS or that a longer treatment period is needed to produce an increase in ferritin levels.

Maitake extract appeared to be safe in this MDS population. We found no evidence of altered disease activity or progression. Mild eosinophilia was noted in four patients, including one patient with associated diarrhea. The etiology and clinical significance of peripheral eosinophilia in this context is unclear and could have been an allergic reaction to the mushroom [41]. Eosinophilia and basophilia together predicted reduced survival without affecting leukemia-free survival in intermediate-2-risk MDS patients [42]. No patient in our study showed increased basophils.

Our study has some limitations, including single-arm design and the potential for patients to have similar improvements without the intervention. A future study with a non-treatment control arm is needed to rule this out. In addition, due to the number of tests performed, it is possible that some changes between baseline and 12 weeks were due solely to chance, although this type of effect would also have affected the double baseline values and should have reduced detection of treatment differences.

## Conclusions

Maitake beta-glucan consumption improves neutrophil and monocyte function in lower-risk MDS patients. The enhanced ROS response to *E. coli* ex vivo in response to Maitake extract treatment suggests that Maitake may enhance immune responses against bacterial infection in MDS patients. A larger study of longer duration will be required to see if these observed effects will translate into decreased rates of infection.

## Abbreviations

AML: Acute myelogenous leukemia
ANC: Absolute neutrophil count
ANOVA: Analysis of variance
CFU-GM: Colony-forming unit-granulocyte/macrophage
CI: Confidence interval
CTCAE: Common Terminology Criteria for Adverse Events
DHR-123: Dihydrorhodamine
FAB: French–American–British classification
fMLP: N-formylmethionyl-leucyl-phenylalanine
G-CSF: Granulocyte colony-stimulating factor
GM-CSF: Granulocyte–macrophage colony-stimulating factor
HC: Healthy controls
HIV: Human immunodeficiency virus
HPC: Hematopoietic progenitor cells
IL: Interleukin
IPSS: International Prognostic Scoring System
IQR: Interquartile range
IWG: International Working Group
LPS: Lipopolysaccharide
MDS: Myelodysplastic syndromes
MSKCC: Memorial Sloan Kettering Cancer Center
NADPH: Nicotinamide-adenine dinucleotide phosphate
PMA: Phorbol 12-myristate 13-acetate
RBC: Red blood cell
ROS: Reactive oxygen species
SD: Standard deviation
WBC: White blood cell
